# A germline chimeric KANK1-DMRT1 transcript derived from a complex structural variant is associated with a congenital heart defect segregating across five generations

**DOI:** 10.21203/rs.3.rs-3740005/v1

**Published:** 2023-12-13

**Authors:** Silvia Souza Costa, Veniamin Fishman, Mara Pinheiro, Andre Rodrigueiro, Maria Teresa Sanseverino, Paulo Zielinsky, Claudia M. B. Carvalho, Carla Rosenberg, Ana Cristina Victorino Krepischi

**Affiliations:** University of São Paulo; Siberian Branch of the Russian Academy of Sciences; University of São Paulo; Uniscience do Brasil; Pontifícia Universidade Catolica do Rio Grande do Sul; Federal University of the Rio Grande do Sul; Pacific Northwest Research Institute; University of São Paulo; University of São Paulo

**Keywords:** congenital pulmonary and aortic valvular stenosis, pulmonary artery stenosis, structural variation, germline quimeric transcripts, KANK1

## Abstract

Structural variants (SVs) pose a challenge to detect and interpret, but their study provides novel biological insights and molecular diagnosis underlying rare diseases. The aim of this study was to resolve a 9p24 rearrangement segregating in a family through five generations with a congenital heart defect (congenital pulmonary and aortic valvular stenosis, and pulmonary artery stenosis), by applying a combined genomic analysis. The analysis involved multiple techniques, including karyotype, chromosomal microarray analysis (CMA), FISH, whole-genome sequencing (WGS), RNA-seq and optical genome mapping (OGM). A complex 9p24 SV was hinted at by CMA results, showing three interspersed duplicated segments. Combined WGS and OGM analyses revealed that the 9p24 duplications constitute a complex SV, on which a set of breakpoints match the boundaries of the CMA duplicated sequences. The proposed structure for this complex rearrangement implies three duplications associated with an inversion of ~ 2Mb region on chromosome 9 with a SINE element insertion at the more distal breakpoint. Interestingly, this hypothesized genomic structure of rearrangement forms a chimeric transcript of the *KANK1/DMRT1* loci, which was confirmed by RNA-seq on blood from 9p24 rearrangement carriers. Altogether with breakpoint amplification and FISH analysis, this combined approach allowed a deep characterization of this complex rearrangement. Although the genotype-phenotype correlation remains elusive from the molecular mechanism point of view, this study identified a large genomic rearrangement at 9p segregating with a familial congenital clinical trait, revealing a genetic biomarker that was successfully applied for embryo selection, changing the reproductive perspective of affected individuals.

## Introduction

Structural variations (SVs) can have a significant impact on congenital human diseases (Schuy et al. 2022). Duplications at the short arm of chromosome 9 (9p) are frequent autosomal alterations of the newborns (Temtamy et al. 2007; Guilherme et al. 2014; Cammarata-Scalisi 2019), with more than 200 cases reported up to now (Sams et al. 2022). In the majority of the cases, 9p duplication causes global developmental delay and a well-recognized spectrum of findings, such as craniofacial (mainly microcephaly and typical facial dysmorphisms) and hands/toes anomalies, accompanied by a broad spectrum of less common additional varying features, including kidney abnormalities, other skeletal malformations, and congenital heart defects (Sams et al. 2022; Temtamy et al. 2007; Guilherme et al. 2014; Stagi et al. 2014; Morrissette et al. 2003, 9; Nakagawa et al. 1999). This phenotypic heterogeneity can be explained by the large number of genes that can be affected and the variable size of the duplicated regions. In addition, 9p duplications are mostly due to segregation of derivative chromosomes from balanced rearrangements, resulting in additional extra 9p chromosomal abnormalities, and only a few of them are *de novo* pure 9p duplications (Sams et al. 2022; Tkemaladze et al. 2023; Ana Cristina V. Krepischi-Santos and Vianna-Morgante 2003, 9).

Although 9p is a relatively gene-poor genomic segment, it contains more than 450 genes, some of them essential for human development. At least 50 of them were previously associated with human diseases. Several efforts were done to define specific *loci* within 9p responsible for each phenotypic manifestation (Sams et al. 2022; Wilson, Raj, and Baker 1985, 9; Huret et al. 1988), resulting in the delimitation of a minimal critical subregion at 9p24–9p22 (Haddad et al. 1996; Fujimoto, Lin, and Schwartz 1998). However, there is still no consistent genotype-phenotype correlation (Cammarata-Scalisi 2019; Littooij et al. 2002)..

It is noteworthy that both 9p deletions and duplications can be associated with congenital heart defects - CHD (Morrissette et al. 2003, 9; Nakagawa et al. 1999; Sams et al. 2022), implicating one or more *loci* for this pathology in the short arm of chromosome 9. CHD is the most common birth defect in newborns, and a substantive cause of morbidity and mortality in infancy (Houyel and Meilhac 2021).

SVs, including duplications, can affect the expression of genes nearby breakpoints and even several hundred kilobases away (Kabirova et al. 2023). Here we report a complex cryptic rearrangement at 9p24 comprising three duplications, which is segregating in a large pedigree in a dominant pattern through five generations; 22 carriers of the rearrangement were affected by an isolated CHD (pulmonary artery and aortic stenosis). Using a combination of genomic approaches and transcriptomic analysis, we dissect the structure of this complex SV.

## Results

We investigated here a 5-generation family with 22 individuals presenting with a phenotype of non-syndromic pulmonary artery and aortic stenosis.

### Clinical description

#### Clinical description

The proband was followed by a pediatric cardiologist from 2 years of age, with clinical and echocardiographic diagnosis of valvular aortic stenosis and both valvular and supravalvular pulmonary stenosis. At diagnosis, the maximal gradients were 25 mmHg through the aortic valve, 20 mmHg through the pulmonary valve and 15 mmHg at the supravalvular pulmonary artery. The severity of the condition slowly progressed and at the age of 8 the gradients have risen to 34, 70 and 35 mmHg, respectively. He underwent percutaneous dilatation of the pulmonary valve, followed by a progressive clinical worsening of the electrocardiographic, radiological, and echocardiographic signs, with an increase in aortic gradient to 114 mmHg at the age of 15. Cardiac surgery was then indicated, and he was submitted to implantation of a metallic aortic valve prosthesis coupled to supravalvular pulmonary artery angioplasty. Both immediate and late postoperative outcomes were uneventful, and he is still asymptomatic at 42 years of age, with near normal cardiac clinical, radiological, and echocardiographic examinations. The proband’s offspring consisted of three children. The first baby had an early diagnosis of severe valvular aortic and valvular pulmonary stenosis requiring urgent cardiac surgery at 2 months of age; albeit technically successful, it was followed by clinical deterioration and rapid demise. The two other children were unaffected by the CHD and conceived by in vitro fertilization followed by embryo selection, based on the results of the genomic analysis described here.

### G-banded karyotype, whole-exome sequencing and chromosomal microarray analysis (CMA)

G-banded karyotype of the proband showed no abnormalities (**Supplementary Fig. 1a**), and exome sequencing did not detect any pathogenic variants. CMA at 180K resolution (Agilent Technologies) was performed in proband’s DNA sample extracted from peripheral blood, revealing copy number variants (CNVs) of 9p24.3 sequences < 500 kb: two adjacent duplicated genomic segments, interspersed between a normal copy number segment (**Supplementary Fig. 1b**).

**Figure 1:**
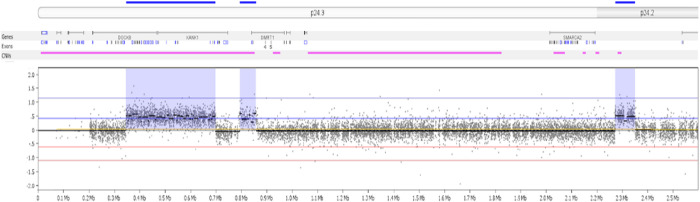
Chromosomal microarray analysis (CMA) revealed a complex pattern of 9p24 duplications The plot shows the copy number profile (log_2_ ratios, Y axis) of the distal region of the short arm of chromosome 9 (9p24), with probes (black dots) depicted according to their genomic coordinates (from pter to the centromere, X axis). To further refine the duplications breakpoints, we applied a CMA (array-CGH) based on a custom 44K (Agilent) platform covering at higher resolution the 9p sequences, which confirmed the presence of two adjacent 9p24.3 duplications (**dup1** and **dup2**) and disclosed a third (**dup3**) one at 9p24.3 (blue shadows and dark blue horizontal lines in the 9p24 ideogram). Above the CNV plot, regions with polymorphic CNVs are presented (pink horizontal lines), as well as genes mapped to the segment (black lines) with respective exons. Image extracted from Nexus Copy Number software (Bionano).

To further refine the breakpoints of the duplicated sequences, a high-resolution 9p microarray was used, which delimited the two 9p24.3 duplications and disclosed a third duplicated segment mapped to 9p24.2 (Fig. 1). The genomic coordinates of the three duplicated segments were as follows: **dup1**: arr[GRCh38] 9p24.3(346084_700056)x3, a 354,973 bp segment with partial duplication of *DOCK8* and *KANK1* sequences (OMIM morbid genes); **dup2**: arr[GRCh38] 9p24.3(788363_864099)x3, a 75,737 bp segment with partial *DMRT1* duplication (not a OMIM morbid gene); and **dup3**: arr[GRCh38] 9p24.2 (2271746_2351089)x3, a 79,344 bp region (no genes), centromeric to the *SMARCA2* gene.

Although the duplicated segments partially encompass *DOCK8, KANK1* and *DMRT1* sequences, the resulting structure could not lead to gene disruption. Moreover, this region is covered by several overlapping CNVs (duplications and deletions) in control populations (DGV - http://dgv.tcag.ca/dgv/app/home). Therefore, the 9p24 CNVs were classified as variants of unknown significance (VUS).

**Figure 2:**
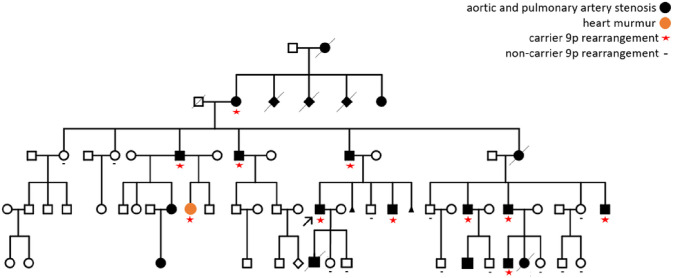
Family pedigree showing five generations of individuals affected by an isolated congenital heart disease The arrow indicates the proband (black symbols denoted affected individuals with pulmonary artery and aortic stenosis; orange is a female patient who was born only with heart murmur). All affected individuals evaluated by CMA were carriers of the 9p24 rearrangement (red asterisk), while evaluated normal family members were non-carriers (black minus symbol). The two alive affected individuals of the last generation were not tested. The two unaffected children of the proband were conceived by *in vitro* fertilization followed by embryo selection, based on the results of the genomic analysis described here.

The proband is one of the 22 affected individuals of a large family with aortic and pulmonary artery stenosis transmitted in a dominant pattern through five generations (Fig. 2). To verify a possible association of the complex SV detected in the proband with the phenotype, 21 additional family members were evaluated by CMA (data not shown). The analysis revealed that the 9p24 rearrangement segregated with the CHD in all 11 affected individuals, while it was absent in all 11 normal relatives.

### Whole-genome sequencing (WGS) and optical genome mapping (OGM)

#### To dissect the structure of the 9p chromosomal rearrangement discovered by CMA, we employed WGS and OGM techniques.

**Figure 3:**
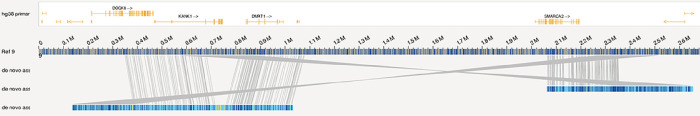
Optical genome mapping reveals the structure of the 9p24 complex rearrangement Single molecules view of proband DNA sample mapping to reference chromosome 9 with breakpoints mapped to *DOCK8, KANK1* and *DMRT1*. GRCh38 reference chromosomes with OGM label patterns are shown in green, and assembled maps of hybrid molecules with label patterns are shown in light blue. Alignments between reference maps and hybrid molecules are shown as gray strings. Overlapping genes are depicted as orange bars. Image extracted from Acess software.

OGM at 100X coverage was analyzed regarding the 9p24 rearrangement and other possible SVs. Although the duplications could be visualized in the copy number track, they were not called by either Access software pipelines (pipeline CNV, which detects copy number changes > 500 kb; and pipeline SV, which detects duplications > 30kb). Analysis of the detected SVs showed the presence of three hybrid molecules at 9p24 (Fig. 3). These 9p24 hybrid molecules were manually analyzed based on the genomic coordinates at the breakpoints/junction sequences, identifying breakpoints and an inversion.

**Figure 4:**
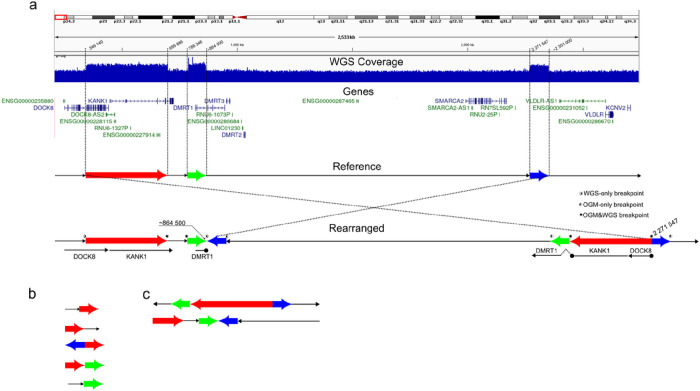
Assembling the complex 9p24 chromosome rearrangement a. IGV screenshot showing WGS read coverage, genes, and breakpoint coordinates in 9p regions. The reference sequence and proposed rearrangement structure are shown below the screenshot, with duplicated segments depicted as colored arrows (red for dup1, green for dup2, and blue for dup3). Schematic representation of the breakpoints (represented by *) identified using WGS (b) and OGM (c) data.

WGS data analysis identified the three 9p24 duplicated segments, showed two breakpoints with discordant reads, and indicated that both homologs of chromosome 9 contain at least one copy of the concordant sequence around the breakpoints, as shown in Fig. 4 (a and b). OGM data confirmed all the breakpoints identified by WGS analysis and revealed one additional junction, including an inverted segment.

Taken together, WGS and OGM analyses confirmed the three 9p24 duplications reported by CMA, disclosed an SV complex pattern, and identified a partially overlapping set of breakpoints (Fig. 4), all of them matching the boundaries of the CMA duplicated sequences.

The combined evaluation of data allowed us to propose a structure for this complex rearrangement, which implies an inversion of ~ 2Mb region on chromosome 9 with partial duplications at the breakpoint regions. Since this 2 Mb inversion is confirmed, this structure looks like a large inverted region with duplicated and non-duplicated sequences.

To validate the proposed 9p24 rearrangement structure, we amplified and sequenced by Sanger the breakpoint, **dup2-dup3** which was not covered by split-reads in the WGS data. Due to the presence of homopolymer tracts within these regions, we were not able to obtain complete end-to-end sequence. Thus, we employed NGS to analyze the obtained amplicons. This analysis confirmed the junction between dup2 and inverted dup3 regions and revealed an insertion of a ~ 500 bp sequence between them that corresponds to a SINE element (**Supplementary Fig. 2**).

*FISH* analysis with BAC probes (**Supplementary Fig. 3**) mapped to 9p24.3 duplicated segment **dup1** showed that the additional genomic copies were not inserted in other chromosomes or moved to a distant region of chromosome 9. In addition, the rearranged chromosome 9 appeared to carry an inverted segment inserted between the two duplicated regions **dup1** and **dup2**. We also performed FISH analysis with non-duplicated probes proximal **dup3** to and within the inverted segment, respectively; the results also confirmed the presence of an inversion near dup3.

At this point, 9p24 SV was considered a biomarker for the proband segregating with the phenotype, and this information was used for embryos selection in preimplantation genetic diagnosis (PDG), after fertilization *in vitro* (IFV). Two healthy non-carrier children were born after this procedure.

### RNASeq analysis and splicing prediction using AI

**Figure 5:**
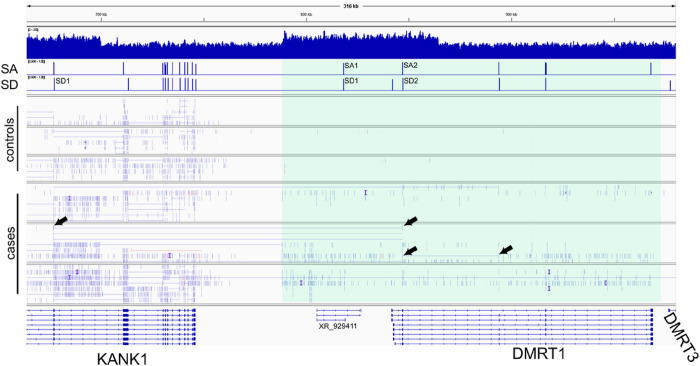
Transcriptome data analysis in blood suggesting the formation of *KANK1-DMRT1*chimeric transcripts The top track shows genomic coverage for two duplicated segments, dup1 and dup2. SA and SD tracks show probabilities of splice-acceptor (SA) and splice-donor (SD) sites inferred using the GENA tool (Fishman et al. 2023). The highlighted region (pale green) shows RNA-seq reads detected only in cases, and not in controls. The pairs of arrows above and below indicate, respectively, examples of reads supporting exon junctions of *DMRT1* and *KANK1*, and only *DMRT1*exons.

The proposed structure of the 9p24 rearrangement suggests the formation of a chimeric transcript, which would include a 5’-prime fragment of *KANK1* (exons 1 and 2 of the MANE-annotated isoform of *KANK1* ENST00000382297.7) and a portion of *DMRT1* locus. To validate this hypothesis, we performed a RNA-seq experiment on blood samples from three 9p24 rearrangement carriers and three unrelated controls. In all samples, we observed high *DOCK8* expression and low-level expression of *KANK1* (Fig. 5). In control samples, we detected no RNA-seq reads aligning to the *DMRT1* locus, consistent with the testis-specific expression pattern of this gene (Raymond et al. 1999). However, in the 9p24 SV carriers, the duplicated region including *DMRT1* gene was covered by RNA-seq reads. All reads mapped in this region support transcription from the forward strand; this strand should be observed if transcription starts from the *KANK1* gene promoter.

To predict the structure of the chimeric transcript which can be formed at *KANK1-DMRT1* breakpoint, we infer splice acceptor (SA) and donor (SD) sites using GENA tool (Fishman et al. 2023). Within *KANK1* and *DMRT1* genes, the predicted splice sites match known exon junctions. As shown in Fig. 5, the *KANK1* gene is truncated after splice-donor site SD1.

Within the region which is presumably transcribed in the rearranged sequence, GENA annotates one pair of SD and SA sites, suggesting the formation of an exon containing 48 nucleotides (ag-GTA CCT ACG CTT GGA AGT GCC AGC ACT ATT ACG TTT CAC TCT GAA CAG-gt). The next splice donor corresponds to the beginning of *DMRT1* exon 2. Thus, the predicted transcript includes the first two *KANK1* exons, 48 bases of additional sequence, and *DMRT1* exons 2–9. The inserted 48-nucleotides sequence contains a stop-codon in the *KANK1* reading frame (*Supplementary material*); thus, the predicted chimeric transcript contains a premature stop-codon, and probably undergoes nonsense-mediated RNA decay. Moreover, *KANK1* exon 2 and *DMRT1* exons are in different reading frames, thus even if the additional 48 nucleotides are not included, the resulting chimeric transcript likely does not encode a functional protein.

Although the coverage of this region is low, we were able to detect several RNA-seq reads confirming junctions of DMRT1 exons and the junction of the *KANK1* exon 2 with *DMRT1* exon 2. Altogether, with breakpoint amplification and FISH analysis, these data confirm the proposed structure for the 9p24 complex SV, indicating that there is an additional copy of *KANK1*, which is truncated, and there is a read-through from this copy into the *DMRT1* locus.

## Discussion

Complex rearrangements are still an underestimated cause of genetic diseases, and in some *loci* they constitute up to 30% of the pathogenic CNVs (Schuy et al. 2022). Sensitivity of the available methods for SV detection is especially limited for resolving complex SVs involving multiple chromosomal segments. This study confirms the importance of a multiomics approach and a combination of different techniques like CMA, FISH, WGS, OGM and RNASeq to fully dissect a complex chromosomal rearrangement. CMA revealed the duplications, whereas WGS/OGM allowed the refinement of the breakpoints, revealed the presence of an inversion, phasing of the multiple rearrangements *in cis,* and provided a framework for the proposal of genomic structure. Although the complex nature of the 9p24 SV was revealed by OGM, confirming breakpoints already detected by WGS and revealing a new one, the duplicated segments were not called, which revealed a limitation of the system. FISH was crucial to show that the duplicated segments mapped on 9p24, and also to support the proposed structure of the rearrangement, with an inversion associated with duplications. Finally, RNA-seq provided experimental evidence of chimeric *KANK1/DMRT1* transcripts, and *in silico* AI-based predictive tools assisted in analysis of the chimeric transcript structure.

Duplication/deletions restricted to the 9p24.3 cytoband, including *DOCK8* and *KANK1*, have been reported across multiple neurodevelopmental/psychiatric phenotypes (Capkova et al. 2021; Glessner et al. 2017). *DOCK8* biallelic mutations cause a recessive condition (https://omim.org/entry/243700); its disruption in heterozygosity was identified in a few patients with mental retardation and/or seizures (Griggs et al. 2008), who were not further evaluated by the presence of additional pathogenic variants by exome analysis. This is the case for several reports of 9p24.3 CNV cases, and current data can only support a possible contribution to neurodevelopmental/psychiatric phenotypes in a multifactorial model. Therefore, an association of 9p24.3 heterozygous CNVs with clinical findings, as major variants with high impact, is still controversial. CNVs encompassing *DOCK8* or *KANK1* are detected in the general population at a relatively high frequency, and an eventual contribution to a congenital rare phenotype should be evaluated with caution. The absence of a neurodevelopment phenotype associated with the *DOCK8/KANK1* duplication (**dup1**) disclosed in our family is not surprising.

Haploinsufficiency of *DMRT1*, 2 and 3, mainly due to 9p24.3 deletions, were already associated with disorders of the sexual development, such as ambiguous external genitalia in males, as well as gonadal dysgenesis (OMIM #154230 46XY sex reversal 4; (Muroya et al. 2000; Shan et al. 2000; Livadas et al. 2003; Quinonez et al. 2013)). In the current case, there is involvement only of the *DMRT1* gene (**dup2**), and similar phenotypes are not present in the 9p24 SV carriers reported here. In association with the duplications and inversion, we detected a non-reference (both GRCh38 and T2T) SINE insertion at one of the breakpoints, disrupting one of the copies of the *DMRT1* gene. SINE is a transposable element and its mobilization has long been associated with evolution and human diseases (Akrami and Habibi 2014; Pfaff, Singleton, and Kõks 2022). Several cases linked with SINE-VNTR-Alus rearrangements induce aberrant splicing patterns, and we cannot exclude the possibility that this insertion alters the *DMRT1* expression pattern. Copy number variants overlapping the short arm of chromosome 9 were already associated with CHD (ref), implicating one or more *loci* in this genomic region. The genetic landscape of CHD is complex, and an interesting emerging feature is that CHD mutations often alter gene/protein dosage (Fahed et al. 2013; Simmons and Brueckner 2017; Yasuhara and Garg 2021). There are several genes mapped to the short arm of chromosome 9 that have been associated with CHD, such *KANK1* (Nguyen and Lee 2022; Botos et al. 2023; Hensley et al. 2016), *SMARCA2* (Lim, Foo, and Chen 2021; Wang et al. 2022), *IFT74* (Bakey et al. 2023), *PIGO* (Krawitz et al. 2012), *DNAI1* (Kennedy et al. 2007; Nakhleh et al. 2012), and *NFIB* (Rao and Goel 2020; Schanze et al. 2018). *KANK1* and *SMARCA2* are involved in the studied SV, respectively in **dup1** and in the inversion.

*SMARCA2* is not disrupted by the rearrangement, but it is included in the inverted segment. The haploinsufficiency of *SMARCA2* causes two dominant developmental conditions, namely Blepharophimosis-impaired intellectual development syndrome (OMIM #619293) and Nicolaides-Baraitser (OMIM #601358), with other clinical signs including CHD. However, as both conditions are associated with severe syndromic intellectual disability, it is not probable that its expression is disrupted by the rearrangement.

Regarding *KANK1*, deletion of the paternal allele was reported in one single family to cause the condition named cerebral palsy, spastic quadriplegic 2 (OMIM #612900); however, no following studies support this association. Indeed, chromosome 9 uniparental disomy is not related to imprinted syndromes (Elbracht et al. 2020), and clinical findings in UPD(9) are commonly attributed to homozygous variants in genes related to recessive conditions or residual trisomy in mosaic. Currently, there is no clinical evidence for haploinsufficiency or triplosensitivity of *KANK1* (KANK1 curation results for Dosage Sensitivity). Notwithstanding, we have found evidence in literature proposing a role for *KANK1* in cardiac development (Nguyen and Lee 2022; Botos et al. 2023). *KANK* genes are scaffold proteins, bridging microtubules to focal adhesion sites (Botos et al. 2023; Pan et al. 2018). The Kank1 protein expression was shown to be widely distributed in various murine tissues, with relatively high levels in cardiac muscle (Nguyen and Lee 2022). In humans, the longest transcript (NM_015158) shows tissue specific expression, predominantly in heart and kidney. In addition, it was found in an injury-specific gene regulatory network in a transcriptome analysis related to cardiac regeneration in the zebrafish (Botos et al. 2023).

It is not clear how a complex SV involving three DNA segments was formed, with six breakpoints (two in each CNV) with three breakpoint junctions. At both sides flanking the dup2-dup3 breakpoint, we observed microhomologies of simple repeats composed of polyA/T sequences. However, insertion of a non-reference SINE element between dup2 and dup3 argues against non-allelic recombination caused by homology of these polyA/T sequences. Alternatively, the SINE insertion might be present in the ancestral chromosome on which the rearrangement took place or an additional event occurring after SV has been formed. It is interesting to note that the transcriptome analysis detected the presence of a chimeric transcript encompassing *KANK1* and *DMRT1* exons, maybe reinforcing a modified product of *KANK1* as a candidate for the phenotype. The role of chimeric transcripts as cause of congenital defects is poorly explored (Zuccherato et al. 2016), in contrast to fusion transcripts commonly described as somatic events in cancer (Salokas, Dashi, and Varjosalo 2023). Only isolated cases were reported related to the detection of chimeric transcripts (gene fusions) as underlying molecular cause of developmental/neurological phenotypes (Boone et al. 2014; Ferrari et al. 2017). Recently, two studies employed an approach of detecting chimeric transcripts using RNA-seq data in rare congenital diseases, one of them with individuals with birth defects (Yamada et al. 2021; Oliver et al. 2019), leading to an increased diagnostic rate. However, *in silico* analysis in the current case predicted a premature stop-codon in the fusion transcript, which probably would undergo nonsense-mediated RNA decay. An eventual contribution of this fusion *KANK1-DMRT1* gene to the cardiac phenotype remains to be fully explored.

Considering the recent report of ultra-long-range interactions between active regulatory elements (Friman et al. 2023), distant 9p genes with normal copy number could be misregulated due to this 9p rearrangement, which makes the derivation of genotype-to-phenotype association relationships even more complicated. In particular, the study of this SV was crucial for genetic counseling and reproductive choices of the family. Even without the identification of the precise mechanism underlying the CHD phenotype, this study identified the SV as a biomarker that was used to identify embryos at risk and select for implantation those without the CHD risk. This strategy resulted in a healthy offspring for at least one couple.

## Patients and Methods

### Patients and genomic samples

Written informed consent for this study was obtained from affected individuals or their parents. Genomic DNA samples were extracted from peripheral blood of 22 family members (n = 11 patients and n = 11 non-affected relatives), using standard procedures (phenol-chloroform followed by ethanol precipitation). RNA samples were obtained from peripheral blood of three male patients and three non-related male controls using the RNeasy Mini Kit (QIAGEN).

### GTG-banded karyotype, FISH and chromosomal microarray analysis (CMA)

Peripheral blood temporary culture (72h) was performed in the presence of phytohemagglutinin, and GTG-banding was obtained according to standard methods. FISH analysis based on metaphase spreads and interphase preparations was performed using BAC clones, as previously described (A. C. V. Krepischi-Santos et al. 2009), with genomic sequences mapped to the short arm of chromosome 9.

Chromosome microarray analysis (array-CGH) was done according to the manufacturer’s instructions, using a 180K and a custom tiling-path high-resolution chromosome 9p oligonucleotide platform (Agilent Technologies, (Grochowski et al. 2018)). Data were extracted and analyzed for copy number changes using the software Nexus Copy Number Discovery (Bionano) and analyzed as previously reported (Krepischi et al. 2022). CNV calls were based on at least three consecutive probes with aberrant log_2_ ratios and compared with the Database of Genomic Variants (http://projects.tcag.ca/variation/) aiming to exclude polymorphisms (frequency > 1%). Detected CNVs were classified according to five tiers of pathogenicity, following the guidelines suggested by (Riggs et al. 2020). The following public databases were consulted: University of California Santa Cruz (UCSC) (http://genome.ucsc.edu/), OMIM (https://omim.org/), ClinGen Dosage Sensitivity (https://search.clinicalgenome.org/kb/gene-dosage/cnv?page=1&size=25&search=), DECIPHER (https://www.deciphergenomics.org/), and PubMed (https://pubmed.ncbi.nlm.nih.gov/).

### Whole-genome sequencing (WGS) analysis

WGS data of six individuals (carriers of the 9p rearrangement, and three unrelated controls) was obtained. Briefly, genomic libraries were constructed with 1 μg of genomic DNA and sequenced on the Illumina HiSeq 2500 platform using 150 base paired end reads (~ 30x coverage). Reads were aligned to the GRCh38 human genome reference using the BWA algorithm (Li 2013) to generate the BAM files, and PCR duplicates were removed from further investigation by Picard tools (v.1.8, http://broadinstitute.github.io/picard/). The Genome Analysis Toolkit (GATK 3.7) (McKenna et al. 2010) was used to realign indels, recalibrate the bases, and call (Unified Genotyper) and recalibrate variants (VQSR).

For analysis, based on the alignment results, we computed tracks showing the depth of coverage (using deepTools bamCoverage) (Ramírez et al. 2016) and discordant read pairs (using samtools) (Danecek et al. 2021). These data were visualized using the IGV software. Breakpoint structures were assessed based on the following filters: the presence of split-reads ( > = 4 in case, no reads in control), with matching supplementary segment sequence; and the presence of discordantly aligned mates ( > = 5 in case, <=1 in control), with matching mate alignment coordinates. The orientation of the DNA segments was determined based on the alignment locations and strands. For two breakpoints where discordant reads were detected, we identified single nucleotide variants (SNV) near the breakpoints. Analyzing SNV distribution in reads, we classified all pairs as: 1) concordant read pair with reference sequence; 2) discordant read pair with an alternative sequence; 3) concordant read pair with an alternative sequence. These data indicate that both homologs of chromosome 9 contain at least one copy of the concordant sequence around the breakpoint, as shown in the first two lines in Fig. 4B.

### Optical genome mapping (OGM) data analysis

OGM was conducted with ultra-high molecular weight DNA samples (> 150 kb) extracted from peripheral blood cells of the proband using the Bionano Prep SP Blood and Cell DNA Isolation kit (Bionano, San Diego, CA, USA). DNA labeling was performed using the DLS DNA Labeling Kit (Bionano, San Diego, CA, USA) to add fluorophores to the specific motif “CTTAAG,” and the sample was run on the Saphyr chip to collect data on the Saphyr System (Bionano, San Diego, CA, USA) at 100x coverage. OGM data were analyzed using the De Novo Assembly pipeline, followed by CNV and SV pipelines, and visualized using the Bionano Access software.

### RNA-seq analysis

Total RNA samples extracted from peripheral blood of three patients and three unrelated male controls were used to build cDNA libraries using the TruSeq^®^Stranded Total RNA LT- kit (with Ribo-Zero TM Gold) (Illumina, USA). Sequencing was performed on the NextSeq 500 platform Mid Output v2 Kit (150 cycles) (Illumina, USA). The FASTQ files were aligned against the ribosomal reference sequence (NCBI, 12/2017) using the BWA software [26] version 0.7.17-r1188, in MEM mode, with the standard parameters, except for the -t 4 parameters. Reads not aligned to ribosomal sequences went to the alignment step against the reference sequence of the human genome (version GRCh37 - hg19) using the STAR software [27], version 2.6.1a_08–27. The annotation database (GTF file) used was the Ensembl file in version 87 in the same build as the human genome reference (GRCh37).

### Breakpoint amplicon sequencing

One of the breakpoints was amplified by PCR using proband’s and control genomic DNA as template (94°C, 4 min; [94°C, 30 sec; 67°C, 40 sec; 72°C, 2 min)×14 cycles, decreasing the annealing temperature by 0.5°C after each cycle; (94°C, 30 sec; 60°C, 40 sec; 72°C, 2 min) × 23 cycles; 72°C, 10 min; 4°C). Primer sequences were 9pD3_2350050F:tgaggtcaagcatctttttatatg, 9pD2_863684F:cgtcagatttcggacccaca, and 9pD2_863969F: gtgcaagtttcccccGACTA. Libraries of the two amplicons were prepared using E6177S NEBNext ultra II FS library preparation with beads and E7710S NEBNext multiplex oligos for illumina set 3 (index CACTCA and CAGGCG) and sequenced on an Illumina MiSeq equipment with MiSeq Reagent Nano Kit v2 (300-cycles). The total covered genomic region was ~ 2 Mb. Sequence reads were aligned to the reference human genome (hg37).

## References

[1] Akrami Seyed Mohammad, and Habibi Laleh. 2014. “Retrotransposons and Pediatric Genetic Disorders: Importance and Implications.” Journal of Pediatric Genetics 3 (1): 9–16. 10.3233/PGE-14081. 27625862 PMC5020984

[2] Bakey Zeineb, Cabrera Oscar A., Hoefele Julia, Antony Dinu, Wu Kaman, Stuck Michael W., Micha Dimitra, 2023. “IFT74 Variants Cause Skeletal Ciliopathy and Motile Cilia Defects in Mice and Humans.” PLoS Genetics 19 (6): e1010796. 10.1371/journal.pgen.1010796. 37315079 PMC10298753

[3] Boone Philip M., Yuan Bo, Campbell Ian M., Scull Jennifer C., Withers Marjorie A., Baggett Brett C., Beck Christine R., 2014. “The Alu-Rich Genomic Architecture of SPAST Predisposes to Diverse and Functionally Distinct Disease-Associated CNV Alleles.” American Journal of Human Genetics 95 (2): 143–61. 10.1016/j.ajhg.2014.06.014. 25065914 PMC4129405

[4] Botos Marius Alexandru, Arora Prateek, Chouvardas Panagiotis, and Mercader Nadia. 2023. “Transcriptomic Data Meta-Analysis Reveals Common and Injury Model Specific Gene Expression Changes in the Regenerating Zebrafish Heart.” Scientific Reports 13 (April): 5418. 10.1038/s41598-023-32272-6. 37012284 PMC10070245

[5] Cammarata-Scalisi Francisco. 2019. “Trisomy 9p. A Brief Clinical, Diagnostic and Therapeutic Description.” Archivos Argentinos De Pediatria 117 (5): e473–76. 10.5546/aap.2019.eng.e473. 31560494

[6] Capkova Zuzana, Capkova Pavlina, Srovnal Josef, Adamova Katerina, Prochazka Martin, and Hajduch Marian. 2021. “Duplication of 9p24.3 in Three Unrelated Patients and Their Phenotypes, Considering Affected Genes, and Similar Recurrent Variants.” Molecular Genetics & Genomic Medicine 9 (3): e1592. 10.1002/mgg3.1592. 33455084 PMC8104183

[7] Danecek Petr, Bonfield James K., Liddle Jennifer, Marshall John, Ohan Valeriu, Pollard Martin O., Whitwham Andrew, 2021. “Twelve Years of SAMtools and BCFtools.” GigaScience 10 (2): giab008. 10.1093/gigascience/giab008. 33590861 PMC7931819

[8] Elbracht Miriam, Binder Gerhard, Hiort Olaf, Kiewert Cordula, Kratz Christian, and Eggermann Thomas. 2020. “Clinical Spectrum and Management of Imprinting Disorders.” Medizinische Genetik 32 (4): 321–34. 10.1515/medgen-2020-2044. PMC11080600 38836202

[9] Fahed Akl C., Gelb Bruce D., Seidman J. G., and Seidman Christine E.. 2013. “Genetics of Congenital Heart Disease: The Glass Half Empty.” Circulation Research 112 (4): 10.1161/CIRCRESAHA.112.300853. 10.1161/CIRCRESAHA.112.300853. PMC3827691 23410880

[10] Ferrari Luca, Scuvera Giulietta, Tucci Arianna, Bianchessi Donatella, Rusconi Francesco, Menni Francesca, Battaglioli Elena, Milani Donatella, and Riva Paola. 2017. “Identification of an Atypical Microdeletion Generating the RNF135-SUZ12 Chimeric Gene and Causing a Position Effect in an NF1 Patient with Overgrowth.” Human Genetics 136 (10): 1329–39. 10.1007/s00439-017-1832-5. 28776093

[11] Fishman Veniamin, Kuratov Yuri, Petrov Maxim, Shmelev Aleksei, Shepelin Denis, Chekanov Nikolay, Kardymon Olga, and Burtsev Mikhail. 2023. “GENA-LM: A Family of Open-Source Foundational Models for Long DNA Sequences.” bioRxiv. 10.1101/2023.06.12.544594. PMC11734698 39817513

[12] Friman Elias T., Flyamer Ilya M., Marenduzzo Davide, Boyle Shelagh, and Bickmore Wendy A.. 2023. “Ultra-Long-Range Interactions between Active Regulatory Elements.” Genome Research 33 (8): 1269–83. 10.1101/gr.277567.122. 37451823 PMC10547262

[13] Fujimoto A., Lin M. S., and Schwartz S.. 1998. “Direct Duplication of 9p22-->p24 in a Child with Duplication 9p Syndrome.” American Journal of Medical Genetics 77 (4): 268–71. 9600733

[14] Glessner Joseph T., Li Jin, Wang Dai, March Michael, Lima Leandro, Desai Akshatha, Hadley Dexter, 2017. “Copy Number Variation Meta-Analysis Reveals a Novel Duplication at 9p24 Associated with Multiple Neurodevelopmental Disorders.” Genome Medicine 9 (1): 106. 10.1186/s13073-017-0494-1. 29191242 PMC5709845

[15] Griggs Bradley L., Ladd Sydney, Saul Robert A., DuPont Barbara R., and Srivastava Anand K.. 2008. “Dedicator of Cytokinesis 8 Is Disrupted in Two Patients with Mental Retardation and Developmental Disabilities.” Genomics 91 (2): 195–202. 10.1016/j.ygeno.2007.10.011. 18060736 PMC2245991

[16] Grochowski Christopher M., Gu Shen, Yuan Bo, Tcw Julia, Brennand Kristen J., Sebat Jonathan, Malhotra Dheeraj, 2018. “Marker Chromosome Genomic Structure and Temporal Origin Implicate a Chromoanasynthesis Event in a Family with Pleiotropic Psychiatric Phenotypes.” Human Mutation 39 (7): 939–46. 10.1002/humu.23537. 29696747 PMC5995661

[17] Guilherme Roberta Santos, Meloni Vera Ayres, Perez Ana Beatriz Alvarez, Pilla Ana Luiza, de Ramos Marco Antonio Paula, Dantas Anelisa Gollo, Takeno Sylvia Satomi, Kulikowski Leslie Domenici, and Melaragno Maria Isabel. 2014. “Duplication 9p and Their Implication to Phenotype.” BMC Medical Genetics 15 (December): 142. 10.1186/s12881-014-0142-1. 25526829 PMC4411943

[18] Haddad B. R., Lin A. E., Wyandt H., and Milunsky A.. 1996. “Molecular Cytogenetic Characterisation of the First Familial Case of Partial 9p Duplication (P22p24).” Journal of Medical Genetics 33 (12): 1045–47. 10.1136/jmg.33.12.1045. 9004142 PMC1050821

[19] Hensley Monica R., Cui Zhibin, Chua Rhys F. M., Simpson Stefanie, Shammas Nicole L., Yang Jer-Yen, Leung Yuk Fai, and Zhang GuangJun. 2016. “Evolutionary and Developmental Analysis Reveals KANK Genes Were Co-Opted for Vertebrate Vascular Development.” Scientific Reports 6 (June): 27816. 10.1038/srep27816. 27292017 PMC4904190

[20] Houyel Lucile, and Meilhac Sigolène M.. 2021. “Heart Development and Congenital Structural Heart Defects.” Annual Review of Genomics and Human Genetics 22 (August): 257–84. 10.1146/annurev-genom-083118-015012. 34061573

[21] Huret J. L., Leonard C., Forestier B., Rethoré M. O., and Lejeune J.. 1988. “Eleven New Cases of Del(9p) and Features from 80 Cases.” Journal of Medical Genetics 25 (11): 741–49. 10.1136/jmg.25.11.741. 3070043 PMC1051577

[22] Kabirova Evelyn, Nurislamov Artem, Shadskiy Artem, Smirnov Alexander, Popov Andrey, Salnikov Pavel, Battulin Nariman, and Fishman Veniamin. 2023. “Function and Evolution of the Loop Extrusion Machinery in Animals.” International Journal of Molecular Sciences 24 (5): 5017. 10.3390/ijms24055017. 36902449 PMC10003631

[23] Kennedy Marcus P., Omran Heymut, Leigh Margaret W., Dell Sharon, Morgan Lucy, Molina Paul L., Robinson Blair V., 2007. “Congenital Heart Disease and Other Heterotaxic Defects in a Large Cohort of Patients with Primary Ciliary Dyskinesia.” Circulation 115 (22): 2814–21. 10.1161/CIRCULATIONAHA.106.649038. 17515466

[24] Krawitz Peter M., Murakami Yoshiko, Hecht Jochen, Ulrike Krüger Susan E. Holder, Mortier Geert R., Chiaie Barbara Delle, 2012. “Mutations in PIGO, a Member of the GPI-Anchor-Synthesis Pathway, Cause Hyperphosphatasia with Mental Retardation.” American Journal of Human Genetics 91 (1): 146–51. 10.1016/j.ajhg.2012.05.004. 22683086 PMC3397269

[25] Krepischi Ana C. V., Villela Darine, da Costa Silvia Souza, Mazzonetto Patricia C., Schauren Juliana, Migliavacca Michele P., Milanezi Fernanda, 2022. “Chromosomal Microarray Analyses from 5778 Patients with Neurodevelopmental Disorders and Congenital Anomalies in Brazil.” Scientific Reports 12 (1): 15184. 10.1038/s41598-022-19274-6. 36071085 PMC9452501

[26] Krepischi-Santos A. C. V., Rajan D., Temple I. K., Shrubb V., Crolla J. A., Huang S., Beal S., 2009. “Constitutional Haploinsufficiency of Tumor Suppressor Genes in Mentally Retarded Patients with Microdeletions in 17p13.1.” Cytogenetic and Genome Research 125 (1): 1–7. 10.1159/000218743. 19617690 PMC2813804

[27] Krepischi-Santos Ana Cristina V., and Vianna-Morgante Angela M.. 2003. “Disclosing the Mechanisms of Origin of de Novo Short-Arm Duplications of Chromosome 9.” American Journal of Medical Genetics. Part A 117A (1): 41–46. 10.1002/ajmg.a.10634. 12548739

[28] Li Heng. 2013. “Aligning Sequence Reads, Clone Sequences and Assembly Contigs with BWA-MEM.” 10.48550/ARXIV.1303.3997.

[29] Lim Tingsen Benson, Foo Sik Yin Roger, and Chen Ching Kit. 2021. “The Role of Epigenetics in Congenital Heart Disease.” Genes 12 (3): 390. 10.3390/genes12030390. 33803261 PMC7998561

[30] Littooij Annemieke S., Hochstenbach Ron, Sinke Richard J., van Tintelen Peter, and Giltay Jacques C.. 2002. “Two Cases with Partial Trisomy 9p: Molecular Cytogenetic Characterization and Clinical Follow-Up.” American Journal of Medical Genetics 109 (2): 125–32. 10.1002/ajmg.10322. 11977161

[31] Livadas Sarantis, Mavrou Ariathni, Sofocleous Chrystalena, Catherine van Vliet-Constantinidou Maria Dracopoulou, and Dacou-Voutetakis Catherine. 2003. “Gonadoblastoma in a Patient with Del(9)(P22) and Sex Reversal: Report of a Case and Review of the Literature.” Cancer Genetics and Cytogenetics 143 (2): 174–77. 10.1016/s0165-4608(02)00849-x. 12781454

[32] McKenna Aaron, Hanna Matthew, Banks Eric, Sivachenko Andrey, Cibulskis Kristian, Kernytsky Andrew, Garimella Kiran, 2010. “The Genome Analysis Toolkit: A MapReduce Framework for Analyzing next-Generation DNA Sequencing Data.” Genome Research 20 (9): 1297–1303. 10.1101/gr.107524.110. 20644199 PMC2928508

[33] Morrissette Jennifer J. D., Ayala Laufer-Cahana Livija Medne, Russell Karen L., Venditti Charles P., Kline Rochelle, Zackai Elaine H., and Spinner Nancy B.. 2003. “Patient with Trisomy 9p and a Hypoplastic Left Heart with a Tricentric Chromosome 9.” American Journal of Medical Genetics. Part A 123A (3): 279–84. 10.1002/ajmg.a.20293. 14608650

[34] Muroya K., Okuyama T., Goishi K., Ogiso Y., Fukuda S., Kameyama J., Sato H., 2000. “Sex-Determining Gene(s) on Distal 9p: Clinical and Molecular Studies in Six Cases.” The Journal of Clinical Endocrinology and Metabolism 85 (9): 3094–3100. 10.1210/jcem.85.9.6771. 10999792

[35] Nakagawa M., Kato H., Aotani H., and Kondo M.. 1999. “Ebstein’s Anomaly Associated with Trisomy 9p.” Clinical Genetics 55 (5): 383–85. 10422813

[36] Nakhleh Nader, Francis Richard, Giese Rachel A., Tian Xin, Li You, Zariwala Maimoona A., Yagi Hisato, 2012. “High Prevalence of Respiratory Ciliary Dysfunction in Congenital Heart Disease Patients with Heterotaxy.” Circulation 125 (18): 2232–42. 10.1161/CIRCULATIONAHA.111.079780. 22499950 PMC3770728

[37] Nguyen Mai Thi, and Lee Wan. 2022. “Kank1 Is Essential for Myogenic Differentiation by Regulating Actin Remodeling and Cell Proliferation in C2C12 Progenitor Cells.” Cells 11 (13): 2030. 10.3390/cells11132030. 35805114 PMC9265739

[38] Oliver Gavin R., Tang Xiaojia, Schultz-Rogers Laura E., Vidal-Folch Noemi, Jenkinson W. Garrett, Schwab Tanya L., Gaonkar Krutika, 2019. “A Tailored Approach to Fusion Transcript Identification Increases Diagnosis of Rare Inherited Disease.” PloS One 14 (10): e0223337. 10.1371/journal.pone.0223337. 31577830 PMC6774566

[39] Pan Wenfei, Sun Kang, Tang Kun, Xiao Qingpin, Ma Chenxue, Yu Cong, and Wei Zhiyi. 2018. “Structural Insights into Ankyrin Repeat–Mediated Recognition of the Kinesin Motor Protein KIF21A by KANK1, a Scaffold Protein in Focal Adhesion.” Journal of Biological Chemistry 293 (6): 1944–56. 10.1074/jbc.M117.815779. 29217769 PMC5808758

[40] Pfaff Abigail L., Singleton Lewis M., and Sulev Kõks. 2022. “Mechanisms of Disease-Associated SINE-VNTR-Alus.” Experimental Biology and Medicine (Maywood, N.J.) 247 (9): 756–64. 10.1177/15353702221082612. PMC9134764 35387528

[41] Quinonez Shane C., Park John M., Rabah Raja, Owens Kailey M., Yashar Beverly M., Glover Thomas W., and Keegan Catherine E.. 2013. “9p Partial Monosomy and Disorders of Sex Development: Review and Postulation of a Pathogenetic Mechanism.” American Journal of Medical Genetics. Part A 161A (8): 1882–96. 10.1002/ajmg.a.36018. 23824832

[42] Ramírez Fidel, Ryan Devon P., Björn Grüning Vivek Bhardwaj, Kilpert Fabian, Richter Andreas S., Heyne Steffen, Dündar Friederike, and Manke Thomas. 2016. “deepTools2: A next Generation Web Server for Deep-Sequencing Data Analysis.” Nucleic Acids Research 44 (W1): W160–165. 10.1093/nar/gkw257. 27079975 PMC4987876

[43] Rao Anupam Canchi Arun, and Goel Himanshu. 2020. “Pathogenic Nonsense Variant in NFIB in Another Patient with Dysmorphism, Autism Spectrum Disorder, Agenesis of the Corpus Callosum, and Intellectual Disability.” European Journal of Medical Genetics 63 (12): 104092. 10.1016/j.ejmg.2020.104092. 33130023

[44] Raymond C. S., Kettlewell J. R., Hirsch B., Bardwell V. J., and Zarkower D.. 1999. “Expression of Dmrt1 in the Genital Ridge of Mouse and Chicken Embryos Suggests a Role in Vertebrate Sexual Development.” Developmental Biology 215 (2): 208–20. 10.1006/dbio.1999.9461. 10545231

[45] Riggs Erin Rooney, Andersen Erica F., Cherry Athena M., Kantarci Sibel, Kearney Hutton, Patel Ankita, Raca Gordana, 2020. “Technical Standards for the Interpretation and Reporting of Constitutional Copy-Number Variants: A Joint Consensus Recommendation of the American College of Medical Genetics and Genomics (ACMG) and the Clinical Genome Resource (ClinGen).” Genetics in Medicine: Official Journal of the American College of Medical Genetics 22 (2): 245–57. 10.1038/s41436-019-0686-8. 31690835 PMC7313390

[46] Salokas Kari, Dashi Giovanna, and Varjosalo Markku. 2023. “Decoding Oncofusions: Unveiling Mechanisms, Clinical Impact, and Prospects for Personalized Cancer Therapies.” Cancers 15 (14): 3678. 10.3390/cancers15143678. 37509339 PMC10377698

[47] Sams Eleanor I., Ng Jeffrey K., Tate Victoria, Ying-Chen Claire Hou Yang Cao, Lucinda Antonacci-Fulton Khadija Belhassan, 2022. “From Karyotypes to Precision Genomics in 9p Deletion and Duplication Syndromes.” Human Genetics and Genomics Advances 3 (1): 100081. 10.1016/j.xhgg.2021.100081. 35047865 PMC8756500

[48] Schanze Ina, Bunt Jens, Lim Jonathan W. C., Schanze Denny, Dean Ryan J., Alders Marielle, Blanchet Patricia, 2018. “NFIB Haploinsufficiency Is Associated with Intellectual Disability and Macrocephaly.” American Journal of Human Genetics 103 (5): 752–68. 10.1016/j.ajhg.2018.10.006. 30388402 PMC6218805

[49] Schuy Jakob, Grochowski Christopher M., Carvalho Claudia M. B., and Lindstrand Anna. 2022. “Complex Genomic Rearrangements: An Underestimated Cause of Rare Diseases.” Trends in Genetics: TIG 38 (11): 1134–46. 10.1016/j.tig.2022.06.003. 35820967 PMC9851044

[50] Shan Z., Zabel B., Trautmann U., Hillig U., Ottolenghi C., Wan Y., and Haaf T.. 2000. “FISH Mapping of the Sex-Reversal Region on Human Chromosome 9p in Two XY Females and in Primates.” European Journal of Human Genetics: EJHG 8 (3): 167–73. 10.1038/sj.ejhg.5200431. 10780781

[51] Simmons M. Abigail, and Brueckner Martina. 2017. “The Genetics of Congenital Heart Disease… Understanding and Improving Long Term Outcomes in Congenital Heart Disease: A Review for the General Cardiologist and Primary Care Physician.” Current Opinion in Pediatrics 29 (5): 520–28. 10.1097/MOP.0000000000000538. 28872494 PMC5665656

[52] Stagi Stefano, Lapi Elisabetta, Seminara Salvatore, Guarducci Silvia, Pantaleo Marilena, Giglio Sabrina, Chiarelli Francesco, and de Martino Maurizio. 2014. “Long-Term Auxological and Endocrinological Evaluation of Patients with 9p Trisomy: A Focus on the Growth Hormone-Insulin-like Growth Factor-I Axis.” BMC Endocrine Disorders 14 (January): 3. 10.1186/1472-6823-14-3. 24397778 PMC3893409

[53] Temtamy S. A., Kamel A. K., Ismail S., Helmy N. A., Aglan M. S., El Gammal M., El Ruby M., and Mohamed A. M.. 2007. “Phenotypic and Cytogenetic Spectrum of 9p Trisomy.” Genetic Counseling (Geneva, Switzerland) 18 (1): 29–48. 17515299

[54] Tkemaladze Tinatin, Bregvadze Kakha, Papiashvili Nikoloz, Gagua Sopio, and Abzianidze Elene. 2023. “A de Novo Chromosome 9p Duplication in a Female Child with Short Stature and Developmental Delay.” SAGE Open Medical Case Reports 11 (March): 2050313X231160883. 10.1177/2050313X231160883. PMC10031590 36968988

[55] Wang Yuejun Jessie, Zhang Xicheng, Chi Keung Lam Hongchao Guo, Wang Cheng, Zhang Sai, Wu Joseph C., Snyder Michael, and Li Jingjing. 2022. “Systems Analysis of de Novo Mutations in Congenital Heart Diseases Identified a Protein Network in the Hypoplastic Left Heart Syndrome.” Cell Systems 13 (11): 895–910.e4. 10.1016/j.cels.2022.09.001. 36167075 PMC9671831

[56] Wilson G. N., Raj A., and Baker D.. 1985. “The Phenotypic and Cytogenetic Spectrum of Partial Trisomy 9.” American Journal of Medical Genetics 20 (2): 277–82. 10.1002/ajmg.1320200211. 3976721

[57] Yamada Mamiko, Suzuki Hisato, Watanabe Akiko, Uehara Tomoko, Takenouchi Toshiki, Mizuno Seiji, and Kosaki Kenjiro. 2021. “Role of Chimeric Transcript Formation in the Pathogenesis of Birth Defects.” Congenital Anomalies 61 (3): 76–81. 10.1111/cga.12400. 33118233

[58] Yasuhara Jun, and Garg Vidu. 2021. “Genetics of Congenital Heart Disease: A Narrative Review of Recent Advances and Clinical Implications.” Translational Pediatrics 10 (9): 2366–86. 10.21037/tp-21-297. 34733677 PMC8506053

[59] Zuccherato Luciana W., Alleva Benjamin, Whiters Marjorie A., Carvalho Claudia M. B., and Lupski James R.. 2016. “Chimeric Transcripts Resulting from Complex Duplications in Chromosome Xq28.” Human Genetics 135 (2): 253–56. 10.1007/s00439-015-1614-x. 26667017 PMC5485664

